# Implementation effectiveness of health interventions for indigenous communities: a systematic review

**DOI:** 10.1186/s13012-019-0920-4

**Published:** 2019-08-05

**Authors:** Truely Harding, John Oetzel

**Affiliations:** Waikato Management School, University of Waikato, Private Bag 3105, Hamilton, 3240 New Zealand

**Keywords:** Indigenous communities, Health interventions, Non-communicable diseases, Implementation framework, Community engagement

## Abstract

**Background:**

Translating research into practice is an important issue for implementing health interventions effectively for Indigenous communities. He Pikinga Waiora (HPW) is a recent implementation framework that provides a strong foundation for designing and implementing health interventions in Indigenous communities for non-communicable diseases around community engagement, culture-centred approach, systems thinking and integrated knowledge translation. This study addresses the following research question: How are the elements of the HPW Implementation Framework reflected in studies involving the implementation of a non-communicable disease health intervention in an Indigenous community?

**Methods:**

A systematic review was conducted using multiple databases. Studies were included if they involved the implementation or evaluation of a health intervention targeting non-communicable diseases for Indigenous communities in Australia, Canada, New Zealand or the United States of America. Published quantitative and qualitative literature from 2008 to 2018 were included. Methodological appraisal of the included articles was completed using the Joanna Briggs Institute System for the Unified Management, Assessment and Review of Information. Data on the population, topic, methods, and outcomes were detailed for each individual study. Key data extracted included the HPW elements along with study characteristics, who delivered the intervention and health outcomes. Data analysis involved a qualitative synthesis of findings as guided by a coding scheme of the HPW elements.

**Results:**

Twenty-one studies were included. Health topics included diabetes, nutrition, weight loss, cancer and general health. The key themes were as follows: (a) two thirds of studies demonstrated high levels of community engagement; (b) from the culture-centred approach, two-thirds of studies reflected moderate to high levels of community voice/agency although only a third of the studies included structural changes and researcher reflexivity; (c) about a quarter of studies included multi-level outcomes and activities consistent with systems thinking, 40% had individual-level outcomes with some systems thinking, and 33% included individual-level outcomes and limited systems thinking; and (d) almost 40% of studies included high levels of end user (e.g., policy makers and tribal leaders) engagement reflective of integrated knowledge translation, but nearly half had limited end-user engagement.

**Conclusions:**

The HPW Implementation Framework is a comprehensive model for potentially understanding implementation effectiveness in Indigenous communities. The review suggests that the studies are reflective of high levels of community engagement and culture-centredness. The long-term sustainability and translation of evidence to practice may be inhibited because of lower levels of systems thinking and integrated knowledge translation.

**Registration:**

Not registered

**Electronic supplementary material:**

The online version of this article (10.1186/s13012-019-0920-4) contains supplementary material, which is available to authorized users.

## Background

Each year, billions of dollars are spent around the world to support the development of evidence-based health interventions for non-communicable diseases designed to improve human health and reduce health inequities [1, 2]. Only a small fraction of these interventions are ever successfully implemented into practice [3], and efforts to implement these practices can take many years [4]. The translation of evidence-based guidelines into practice is one of the most challenging problems in health care and disease prevention [5]. Despite extensive public health research on the efficacy and effectiveness of health promotion and disease prevention strategies, methods for disseminating these interventions and encouraging their implementation and wide-spread adoption are not well developed or evaluated [5].

Further, little progress has been made in reducing inequities despite the fact that there is strong evidence supporting intervention effectiveness in regards to non-communicable diseases [6]. Researchers acknowledge the need for implementation science and translational research for achieving health equity and have identified key issues including context, culture and levels of acceptance as central to the problem of the utilisation of evidence-based practices [7, 8]. Translation, dissemination, uptake and implementation are becoming increasingly important to transition innovative health research into health policy and practice and ultimately achieve health equity for Indigenous populations [3, 7].

Indigenous populations around the globe face inequities compared to non-Indigenous populations [9, 10]. For example, one study found inequities between Indigenous and non-Indigenous populations in relation to life expectancy, child obesity, adult obesity, educational attainment and economic status [10]. Achieving health equity requires addressing a complex array of contextual and cultural features along with the unjust distribution of social determinants in health rather than simply focusing on intervention efficacy [7, 8]. For example, Indigenous perspectives on holism and wellbeing are based on cultural values, beliefs and traditions passed down the generations, including beliefs in the unity of mind, body and spirit [11]. Indigenous cultures frequently believe that all life is interrelated including the environment and the universe and that holism is the most appropriate way to understand health and wellbeing [12, 13]. Thus, when implementing an intervention with an Indigenous community, the intervention needs to be culturally appropriate and relevant as well as supported and owned by the community [2, 14].

A recent implementation framework provides a strong foundation for understanding the key principles for developing and implementing non-communicable disease health interventions with Indigenous communities. The He Pikinga Waiora (HPW; Enhancing Wellbeing) [15] is a theoretical framework that fills a gap in regards to the lack of implementation models for Indigenous communities, which may help account for the underwhelming progress made in reducing health inequities [16]. HPW is built on a strong international evidence base for best practice in developing and implementing health interventions [15]. Specifically, it argues that implementation science for Indigenous communities should be grounded in Indigenous knowledge, participatory approaches and systems thinking and includes four elements: culture-centred approach, community engagement, systems thinking and integrated knowledge translation.

First, implementation should be guided by the culture-centred approach (CCA). The CCA argues that social structures of health can be transformed by providing opportunities for community voice/agency, reflexivity among researchers, and providing resources to address structural challenges [17, 18]. This transformation is achieved through asserting Indigenous self-determination, challenging power imbalances and health researchers/professionals being reflexive and adjusting their behaviour to enhance cultural safety [15, 19]. Such an approach helps to ensure Indigenous cultural perspectives are part of the definition of the problem and integrated into the interventions to facilitate implementation effectiveness and address health equity [20].

Second, high levels of community engagement (CE) are associated with greater implementation effectiveness and improved health outcomes and health equity [21, 22]. CE is a process of collaborating with groups directly affected by a particular health issue or with groups who are working with those affected [23]. CE ranges from very limited community involvement to community ownership and management through five categories: outreach, consultation, involvement, shared leadership and community-driven [24, 25]. High levels of CE are reflected through shared decision-making and communication among researchers and community members which helps with sustainability, capacity building and long-term health outcomes [26, 27].

Third, systems thinking (ST) helps to address the complexity of the local contexts and the variety of levels and determinants of health problems [28, 29]. ST also facilitates new framings and strategies that are associated with improved project and health outcomes including health equity [29, 30]. It allows for new ways of thinking for researchers, practitioners and community members through considering different perspectives, relationships among people/facets of the health system and multiple level of analysis [30]. ST also acknowledges holistic perspectives towards health problems and examines the inter-relationships of the various parts that need to be understood within a larger context [29]. It is important to note that ST is frequently used and has many different approaches to conceptualising such as complex systems dynamics. As a result, there are no clear guidelines for implementation in practice [31, 32]. The HPW framework specifies key ST elements that may serve as guidelines for implementation of health interventions for Indigenous communities including multiple perspectives, relationships and levels of analysis along with feedback loops.

Finally, integrated knowledge translation (IKT) emphasises co-design and co-production with end users in developing and implementing an intervention for the purpose of transferring knowledge and enhancing sustainability [33, 34]. End users are the people who will use research findings and facilitate the translation from research to practice [35]. These may be clinicians, policy makers, tribal leaders and systems administrators. IKT involves the researchers and end users working in various levels of partnership to ensure there is shared ownership and that many barriers to implementation and translation can be addressed early in the design process [33, 36]. For Indigenous communities especially, IKT also needs to ensure there is benefit for the community reflected in the knowledge of the community [37].

The purpose of this study is to conduct a systematic literature review of articles that involved the implementation of a non-communicable disease health intervention in an Indigenous community. Systematically reviewing the literature will provide insights regarding how the HPW principles are currently being implemented and reported in Indigenous community-based health interventions. This study applies the HPW framework in a post hoc manner to identify the patterns in intervention development and implementation with Indigenous communities.

## Methods

The systematic review was completed using PRISMA guidelines [38] (see Additional file 1 for the checklist). Our primary research question was the following: How are the four elements of the HPW Implementation Framework reflected in studies involving the implementation of a non-communicable disease health intervention in an Indigenous community? This question relies on the post hoc application of the HPW framework to studies that did not directly use it. The rationale for this choice is that there is not an existing framework guiding implementation science of Indigenous health interventions. The HPW framework was recently developed and has a strong theoretical and empirical basis and its post hoc application enables us to examine whether these key elements are being used by researchers and implementers; if so, they can help identify promising practices for researchers and practitioners working in similar communities. If they are not being used, it may illustrate important directions for future research and practice. The HPW framework, previously applied in a post hoc manner, provided insights demonstrating associations between the implementation of framework principles and health outcomes in type 2 diabetes prevention for Indigenous people in primary care [15].

### Inclusion and exclusion criteria

The chosen literature was peer reviewed and published in English since 2008. This time period was selected to provide relatively recent insights to implementation effectiveness and provide a sufficient literature base to review. Literature was only considered if it evaluated and/or implemented a health intervention targeting Indigenous communities. Communities are physical spaces involving Indigenous members who were targeted for benefit from the health intervention. The specific interventions included in this study were those that discussed non-communicable diseases.

The search exclusion criteria eliminated articles that were reviews or editorials. Further, the article was excluded if the intervention took place in a primary health organisation or was based on another aspect of the primary health system. Additionally, school-based interventions were excluded unless the school-based intervention was part of a larger implementation into the community (e.g., involving larger health promotion and community intervention). Literature was excluded if the study population was not Indigenous and if there was no intervention implemented. Literature was also excluded if it only discussed the process of creating and implementing an intervention rather than evaluation of the intervention process and/or outcomes.

### Search strategy

EBSCOhost, Emerald Insight, ProQuest Central, Pubmed and MEDLINE databases were the selected search engines. The key search terms were community health, Māori, First Nation, Aboriginal, Native American, Indigenous and intervention. The search consisted of combining two or three search terms to reveal specific articles that were relevant to the study. The following sequences were the search combinations used for this study: “community health” and “Māori”; “community health” and “Māori” and “intervention”; “community health” and “Indigenous”; “community health” and “Indigenous” and “intervention”; “community health” and “First Nation”; and “community health” and “First Nation” and “intervention”; “community health” and “Aboriginal”; and “community health” and “Aboriginal” and “intervention”; and “community health” and “Native American”; and “community health” and “Native American” and “intervention”. The terms were searched in the article title, abstract, the whole article and the keywords. Literature from each individual search was exported to an EndNote file to identify and eliminate the duplicate articles. Once the duplicates were removed, the study selection process began.

Study selection was completed by the two authors. Titles and abstracts were completed by the first author with consultation with the second if there were uncertainties. The full-text articles were independently reviewed by both authors using the exclusion criteria. After completing the study selection, additional records were identified using three means to locate any missed published or unpublished studies and thus reduce the risk of publication bias [39]. First, a manual search of references from the included articles was undertaken. Second, a search of the grey literature was completed using the search terms in Google. Third, three clinical trial registries (Australia New Zealand Clinical Trials Registry, Health Canada Clinical Trials Database and ClinicalTrials.gov) were searched using key Indigenous-related search terms (e.g., First Nations). Relevant trial descriptions were reviewed; study protocol articles and project names were then searched through Google and Google Scholar to find final study results in published or unpublished form.

### Data extraction and methodological appraisal

From the articles that met the inclusion criteria, the selected data for this study were the population, health topic, methods, measures, outcome(s) of the health intervention, who delivered the intervention and data related to the HPW elements. Given that we are using the HPW framework as a post hoc analysis of the articles and also that some published outcome studies have limited information about intervention development, we also extracted data from cited studies in the included articles such as study protocols, supplemental files, web sites or articles that were referenced to provide more information about the study methods or intervention. In two cases, a follow-up publication identified in study selection helped provide additional information about the primary study [40, 41]. We assigned a rating of the quality of details provided in the articles as good, fair or poor. In all cases, the rating was at least fair with the vast majority rated as good (*n* = 17) which allows the comprehensive assessment of each of the HPW elements (see Additional file 2 for information about additional studies consulted and quality of details).

Methodological appraisal of the included articles was completed using the Joanna Briggs Institute System for the Unified Management, Assessment and Review of Information [42]. The study design for the primary hypotheses/study aims were categorised along with three different types: observational, randomised control trials and qualitative. Each individual study was assessed with risk of bias identified. Appraisal criteria are displayed in Table 1. Each criterion was rated as yes, somewhat, no, unknown or not applicable. An overall score was provided for each study using yes =2, somewhat =1 and no or unknown = 0.

**Table 1:** Methodological appraisal of studies

Observational Studies				Benyshek et al., 2013	Christohper et al., 2008	Coppell et al., 2009	Kaholokula et al., 2014	Kakekagumick et al., 2013	Reilly et al., 2011	Shah et al., 2015
1) Was the study based on a random or pseudo-random sample?				N	Y	Y	N	N	N	N
2) Were the criteria for inclusion in the sample clearly defined?				Y	Y	Y	Y	N	Y	Y
3) Were confounding factors identified and strategies to deal with them stated?				N	N	S	Y	N	Y	N
4) Were outcomes assessed using objective criteria?				Y	N	Y	Y	Y	Y	Y
5) If comparisons are being made, was there sufficient description of the groups?				N/A	N/A	Y	Y	N/A	N/A	N/A
6) Was follow up carried out over a sufficient time period?				S	Y	Y	S	Y	Y	S
7) Were the outcomes of people who withdrew described and included in the analysis?				Y	N	N/A	N	N	N/A	N/A
8) Were outcomes measured in a reliable way?				Y	S	Y	Y	Y	N	Y
9) Was appropriate statistical analysis used?				Y	Y	Y	Y	Y	N	Y
Total				11/16	9/16	15/16	13/18	8/16	8/14	9/14
Randomised Control Trial	Brimblecombe et al. 2017	Canuto et al. 2012	Ho et al. 2008	Kaholokula et al. 2012	Karanja et al. 2010	Kolahdooz et al. 2014	Mendham et al. 2015	Simmons et al. 2008	Sinclair et al., 2013	Tomayko et al. 2016
1) Was the assignment to treatment groups truly random?	Y	Y	N	Y	N	Y	Y	Y	Y	S
2) Were participants blinded to treatment allocation?	Y	N	N	N	N	N	N	N	N	N
3) Was allocation to treatment groups concealed from the allocator?	Y	N	N	N	N	N	N	N	Y	N
4) Were the outcomes of people who withdrew described and included in the analysis?	N/A	Y	N	Y	N	N	N	N	Y	Y
5) Were those assessing outcomes blind to the treatment allocation?	Y	Y	N	U	N	N	N	N	N	N
6) Were the control and treatment groups comparable at entry?	Y	S	Y	Y	Y	Y	Y	Y	Y	Y
7) Were groups treated identically other than for the named interventions?	Y	Y	Y	Y	Y	Y	Y	Y	Y	Y
8) Were outcomes measured in the same way for all groups?	Y	Y	Y	Y	Y	Y	Y	N	Y	Y
9) Were outcomes measured in a reliable way?	Y	Y	S	Y	Y	Y	Y	Y	Y	Y
10) Was appropriate statistical analyses used?	Y	Y	Y	Y	Y	Y	Y	S	Y	Y
Total	18/18	15/20	9/20	14/20	10/20	12/20	12/20	9/20	16/20	13/20
Qualitative Studies							English et al., 2008	Sushames et al. 2017	Townsend et al. 2016	Tumiel Behalter et al. 2011
1) Is there congruency between the stated philosophical perspective between the research and the methodology?							Y	Y	Y	Y
2) Is there congruity between the research methodology and the research question or objectives?							Y	S	Y	S
3) Is there congruity between the research methodology and the methods used to collect data?							Y	Y	S	S
4) Is there congruity between the research methodology and the representation and analysis of data?							S	Y	N	N
5) Is there congruity between the research methodology and the interpretation of results?							Y	Y	Y	S
6) Is there a statement locating the researcher culturally or theoretically?							N	N	N	N
7) Is the influence of the researcher on the research and vice versa addressed?							N	N	N	N
8) Are participants and their voices, adequately represented?							S	Y	Y	N
9) Is the research ethical according to current criteria or, for recent studies, is there evidence of ethical approval by an appropriate body?							S	S	S	S
10) Do the conclusions drawn in the research report flow from the analysis or interpretation of the data?							Y	Y	Y	S
Total							13/20	14/20	12/20	7/20

*Y* Yes, *S* Somewhat, *N* No, *U* Unclear, *N/A* Not applicable; Yes scored two, somewhat one; No and unclear are zero with not applicable removed from totals

### Data synthesis

Summary tables were provided for the primary research question and also for the study characteristics. Data about the HPW elements were described using a coding scheme to guide in the inclusion of key elements [15]. The coding scheme was used to recognise key HPW concepts even if they were not directly labelled as such by the study authors (i.e., current authors’ interpretation of whether HPW elements were used). The key components for each of the elements include the following: (a) CCA—community voice/agency in defining problem and solution/intervention, researcher reflexivity and resources for structural change; (b) CE—degree of shared decision-making and communication among researchers and community entities; (c) ST—multiple perspectives of causes reflecting holism, complex relationships among ideas and entities and multiple levels of analysis (e.g., micro, meso and macro); and (d) IKT—co-design and implementation of intervention with end users. Data were then qualitatively synthesised to provide an overview of how each of the four HPW elements was reflected in the 21 studies.

Data on the population, topic, methods, and outcomes were detailed for each individual study. Individual study results include measures of change and significance values. Given the heterogeneity of study characteristics, measures and outcomes, meta-analysis—or even simple quantitative associations—was not possible. Following a previous systematic review, outcomes were categorised in two ways: (a) having at least one statistically significant change in a primary outcome and (b) having statistically significant changes in 50% or more of the primary outcomes [43]. Additional file 3 presents the study characteristics and outcomes.

## Results

### Study selection

**Fig. 1:**
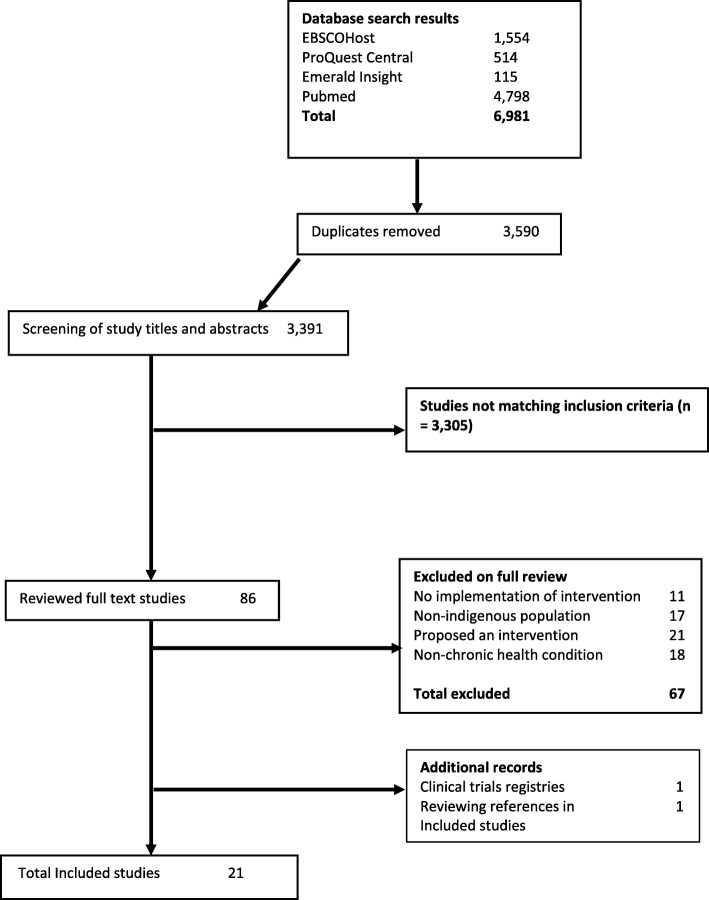
Flowchart diagram detailing the literature search

Figure 1 shows the search strategy identified 6981 articles from the listed databases; articles were downloaded to EndNote to remove a total of 3590 duplicates. Upon screening for inclusion criteria in titles and abstracts, the full text of 86 articles was independently reviewed. After review using the exclusion criteria, 19 articles were included for analysis. The additional record search resulted in two additional articles for a final total of 21.

### Methodological appraisal

Table 1 provides a summary of the results of the appraisal. There were seven observational studies: four from the United States of America (USA) [44–47], one from Australia [48], one from New Zealand [49] and one from Canada [50]. The follow-up period ranged from 4 months to 3 years. Three studies had equal or less than 6 months of follow-up which is a potential area for bias [44, 46, 47]. Retention rates ranged from 55–100% although one study included two independent panels [49] and another did not use individuals as the unit of analysis [48]. The major risk of bias in these studies is the lack of a comparison group although this is consistent with the research design. Additional risks include some lack of valid and reliable measures [45, 48], small sample size [44, 50], lack of appropriate statistical analysis [48], incomplete description of the participants and study methods [50] and lack of information about non-completers [46, 50]. The average study quality rating of observational studies was 68.71% (SD = 15.38).

There were 10 randomised control trials with comparison groups: four from the USA [51–54], three from Australia [55–57], two from Canada [58, 59] and one from New Zealand [60]. Six of the studies randomised individuals to intervention and control/standard care [51, 52, 54, 56, 57, 60], three included random selection of communities to intervention and control [53, 55, 59] and one included selection of communities as well, but it was not clear whether assignment was random [58]. In all but two of the studies, the control group received a delayed intervention [53, 60]. The trial periods ranged from 3–24 months (median 9–12 months) with four studies having less than or equal to 6 months of follow-up [51, 52, 56, 57]. Four studies reported lower than 70% retention rate (59–66%) [54, 56, 57, 60]. The major risks of bias included lack of blinding in randomisation in all but two studies [51, 55], lack of blinding for assessors in all but two studies [55, 56], lack of inclusion of data from those who withdrew (e.g., intention to treat analysis) in all but four studies [51, 52, 54, 56], incomplete reporting of study results in one study [60], some unreliable measures in one study [58] and a few participants moving from one arm to another post-randomisation in one study [54]. The average study quality rating of randomised control trials was 65.00% (SD = 17.16).

There were four qualitative studies: three from the USA [61–63] and one from Australia [64]. Each of these studies described a health intervention and sought to describe the processes by which the programme was developed and how it impacted outcomes [61–63] or how it related to participation in the intervention [62, 64]. Only two studies directly addressed participant outcomes although those included only descriptive information [61, 62]. A risk of bias is that none of the studies included statements of researcher positionality nor did they discuss the influence of the researcher on the research. Further, two of the studies did not provide any direct quotes thus a risk of bias in that participant perspectives were not included [62, 63]. The average study quality rating of qualitative studies was 57.50% (SD = 15.55).

### Study synthesis

The study synthesis addresses the research question about how the HPW elements are reflected in the studies (see Table 2 for the study synthesis). Prior to summarising those findings, Additional file 3 provides a breakdown of the study characteristics that helps to understand the context of the studies. The targeted health conditions included diabetes (48%), obesity/general non-communicable health conditions (24%), nutrition (19%) and cancer (10%). The types of interventions included lifestyle (38%), multi-pronged including individual and community elements (33%), self-management of a condition (14%) and education (14%). Two thirds of the interventions included the delivery of at least one component by a community health worker (CHW). All of the studies that had a measurable outcome variable (*n* = 19) had at least one primary outcome with a statistically significant and improved change with six studies (38%) achieving significant change in 50% of primary outcomes measures. A slight majority of the studies (52%) were feasibility, pilot or short-term interventions.

**Table 2:** Study Synthesis using the He Pikinga Wairoa Elements

Study	Community engagement	Cultural centeredness	Systems thinking	Integrated knowledge translation
Observational Studies				
Benyshek et al. 2013	The intervention was delivered by Native lifestyle coaches and this facilitated connection with participants. The intervention was developed using community-based participatory research (CBPR) methods although specific details on the process are not clear.	Researchers adapted the programme to tailor to urban American Indians. The community was informed and engagement was limited to adapting the intervention; problem definition and solution identification by the community not apparent.	The interview process allowed for feedback loops. The target of change was at the individual level. The systems considered were those that already existed. Limited evidence of systems thinking.	There was no direct discussion with end users other than using CPBR to adapt the intervention.
Christopher et al. 2008	A CBRP approach was used. Strong community involvement at every phase of development and implementation with evidence of shared decision making. Lay health advisors were used to deliver the intervention and were selected by the community.	Both the researcher and community leaders had equal say in the development of the intervention with the community identifying the problem. Intervention was delivered within the structure of the community and provided additional services. Research reflexivity clearly noted in the article.	There was demonstration of good understanding and respecting the community systems. The project altered their intervention based on the feedback received. Messages targeted individuals directly and community indirectly.	Almost every aspect of the intervention was co-created with community members including some community organisations. Engagement with other end users was not directly discussed.
Coppell et al. 2009;	The project had a participatory approach with the community involved in all project phases. A memorandum of understanding (MOU) was established between the researchers and communities before the first attempt of developing a Māori-led diabetes intervention. Community health workers were a key engagement approach with participants.	Researchers completed surveys to assess the severity of diabetes and confirm the communities concerns. The project developed a vision that was shared and owned by the community. The intervention involved multiple structures and provided resources to the community. Researcher reflexivity engaged though the partnership.	The whole community was included. By working with all members of community the intervention impacted the individual, the community and the local stores and businesses, allowing creating opportunities for a positive change at every level. Feedback loops were highlighted as a crucial aspect for this project.	The intervention engaged with stores,schools and local employers to support the intervention structurally (e.g., create school policies consistent with the intervention). The intervention was driven by the community and community leaders. Thus, there was a high-level of engagement with stakeholders and end users.
Kaholokula et al. 2014	The project was part of a CBPR partnership called the PILI ‘Ohana Project. This project was a partnership between researchers with five community organisations. Community members and community organisations were integrated in all phases of the research.	The intervention was adapted with community participation and the community helped identify the problem and solution. Researcher reflexivity reflected in an ongoing partnership. Community peer educators helped to make cultural connection with participants.	Equal partnership of community and academic investigators involved from the beginning of the study to integrate the best combination of community and scientific knowledge. Partnership involved multiple aspects of the Native Hawaiian health system. Only individual level of analysis for the intervention.	The partnership includes relationship with the Native health system, community health centres, and grassroots organisations. The CBPR approach offered the benefit of building capacity within the difficult to reach communities in this study. The level of knowledge translation beyond study period is not described.
Kakekagumick et al., 2013	Research project reflects a long-time partnership involving multiple projects guided by community engagement. Research project included shared decision making and communication in all elements of the research over a 22-year period.	The need for the interventions was identified by the community and the various interventions reflect the wishes of the community. Interventions provided new resources and changed existing structures. Reflexivity is noted through reciprocal learning and capacity building.	A number of projects addressed multiple causes of the health issue, accessibility, costs, environment, social support and lack of adequate facilities. Relationships were forged in each phase with community groups, schools, community health services, and sports teams addressing multiple systems levels. Vast array of feedback was received through these relationships that shaped the design, implementation and outcome of the overall study.	The project is a partnership of the Sandy Lake First Nation and researchers. The project has been active for 22 years demonstrating that it is a sustainable model that has transformed the community over time. End users highly engaged in all phases of the research.
Reilly et al. 2011	The project was overseen by a steering committee of senior community and university representatives. They were bound by a MOU between participating organisations that stipulated rules for community ownership and storage of data. The project was implemented by local health workers. Trust between the university researchers and the Koori community had been established over many years as a result of collaboration on previous projects.	Initial consultation was conducted with the community around the health and health determinants of the community. The academic researchers worked closely with community researchers who coordinated the development and delivery of the program. Academic researchers took a supportive role, offering advice and suggestions but only participating directly in planning and design when requested.	The project created a health promotion programme aligned to an ecological approach. It targeted changes in the nutrition environment with the thought that individual behaviour would change (although not directly assessed in the study). Further, multiple aspects of the health promotion and food supply system were considered.	Community representatives on the steering community were members of Aboriginal partner organisations. Specific activities involved engagement with end users from these organisations. Intervention strategies were devised in response to each aims following discussions between participating organisations and reflections on previous findings and experiences of participants.
Shah et al. 2015	The lead researcher had a prior relationship with the community although there is no clear partnership identified. Tribal leaders and health programs were consulted. Community health representatives recruited participants and delivered the intervention.	The health issue was identified through surveys with community members. Participants were involved in the development of the intervention through focus groups and a cultural specific education interventions. The researchers made the majority of the final design and implementation decisions.	The intervention aimed to deliver healthcare that emphasized greater autonomy/self-management. The intervention targeted individual behaviour and no other levels considered. Limited evidence of systems thinking.	While tribal leaders and health programs were consulted at the beginning, there is no evidence of ongoing engagement with these end users.
Randomised Control Trials				
Brimblecombe et al. 2017	This intervention utilised grocery stores to implement price discounts for healthy food options and provide nutrition education. The community engagement involved finding communities and stores to take part in the intervention. There was an agreement with the non-profit associations affiliated with the stores and store boards. The researchers talked to store boards to share study protocol and sign an agreement to follow the protocol and help recruit research assistants.	That nature of the problem is well defined although the communities did not select the problem directly or suggest the solution. There is general research evidence suggesting Aboriginal communities wanted this type of intervention. The intervention did provide resources (i.e., discounts) to community members to purchase healthy food.	The research focusses on the larger food system as a potential manner to improving healthy food consumption. The overall project includes evaluation of overall food buying patterns for stores and individual behaviour although this study only includes the larger buying patterns. The study recognises the larger systemic issues and relationships that shape individual consumption patterns although the actual study implementation does not directly include engagement with all of these issues and relationships.	Knowledge translation efforts with end users was minimal. The store boards were consulted although only for approval of the study. This is a top-down approach where the researchers have identified an issue and have developed an intervention they feel is appropriate. No discussion of policy or practice change beyond the study period is identified.
Canuto et al. 2012	The project was guided by an advisory committee made up of local Indigenous women. Some of members were representatives from the collaborating organization and provided input into the interpretation of qualitative data. Participants were recruited based on the intervention requirements.	The committee provided advice on feasibility of the project’s procedures and assessments ensuring that all aspects of the project were culturally appropriate. There did not appear to be any direct input on defining the problem although there was input on the solution. Reflexivity not apparent.	The intervention considered a variety of potential factors for programme success. It was focused only on individual level outcomes. Systems levels, relationships and perspectives were not considered at great depth other than barriers for participation.	This intervention development included initial consultation with two community organisations. The focus was more on making sure the intervention was culturally appropriate rather than how to engage in knowledge translation.
Ho et al. 2008	Community engagement was achieved through local organisations helping to facilitate implementation of the project. Decision making and communication was largely directed by the researchers although communities had input that led to adaption of the intervention in the specific locales.	Semi-structured interviews were done with multiple community members to assess acceptability, feasibility and sustainability of the intervention. Thus, some community voice was included to help make the intervention culturally appropriate. There was no direct evidence that the community was able to select the problem to address or how to address it.	This intervention was implemented into three different systems: school, food store and larger community. It planned to change the systems and provide a healthier outlook for both communities. Multiple stakeholders were included and different perspectives gathered. Feedback loops were supplied during feasibility testing.	The majority of the knowledge integrated at the beginning of the project was from the researchers and project team. Community health workers and researchers expressed a desire to sustain the intervention if supplied with materials so there was some potential knowledge translation activities.
Kaholokula et al. 2012	The project was part of a CBPR partnership called the PILI ‘Ohana Project. This project is a partnership between researchers with five community organisations (health centres, health care systems, and grassroots organisations). Compromises were made by each to ensure the best intervention design for the participants providing evidence of shared decision making and communication.	The partnership provided equal insights about the importance of focusing on diabetes. Feedback on the intervention was gathered in three activities; focus groups, informant interviews, and pilot testing. A steering committee was established and oversaw the translation of a previous diabetes intervention to ensure cultural appropriateness. Delivered by community health advocates to enhance cultural connection. Reflexivity evidenced through ongoing partnership.	The study acknowledged the important role community organisations play in designing and implementing health interventions in Indigenous communities. The partnership involved multiple aspects of the Native Hawaiian health system. Only individual level of analysis for the intervention. Systems where altered for the translation of the intervention, though it is unclear if it is an ongoing change, or if it was only for the period of the intervention.	Changes to the intervention were made based off of community organisation advice and these are key end users in the Native Hawaiian health system. No direct discussion of sustainability of the intervention.
Karanja et al. 2010	Researchers worked with various community representatives to craft and implement the intervention including community health workers. Three communities received the intervention and were able to adapt and tailor intervention plans to fit their communities. There was some sharing of decision making and communication through these intervention plans.	Community members and agency representatives were able to help to craft messages and policies. Structural changes were sought in health and business settings. Whether the community had a say in the definition of problem and choice of health issue is not clear despite clear involvement during the intervention development and implementation. Reflexivity is not discussed.	The intervention targeted community and family-level behaviour. The community level include a variety of elements and perspectives including media messages and policy changes in businesses and health settings. There was opportunity for feedback to adapt intervention plans.	Numerous end users were engaged in the implementation of the intervention. They provided responsibility for implementation of aspects of the community intervention. Sustainability or intention to sustain is not discussed.
Kolahdooz et al. 2014	The intervention development process was a community participatory approach through the use of community workshops and qualitative and quantitative formative research. Shared decision making was enacted through the workshops in deciding the appropriate intervention and intervention messages. No mention is made of community researchers or research partners.	The formative research process provided community voice for identifying the problem. The community workshops ensured there was community voice for the solution. Culturally appropriate research processes and interventions were utilized. Reflexivity is not directly discussed.	The intervention process considered multiple perspectives and stakeholders (from grocery stores, community sites and health organisations). Effort was made towards changing the systems that usually promote unhealthy food and eating habits. Relationships among the organisations and larger system were noted in developing a healthy food environment. Intervention targeted community, organisation and individual levels.	Community voice was integrated from the beginning and knowledge translation efforts included participation from community members, local leaders, government health workers and grocery shop staff. There was no mention whether these efforts led to sustainability of the intervention.
Mendham et al. 2015	The intervention was designed and implemented by the project team with minimal engagement from the community. Community organisations were utilised for recruitment of participants. The authors noted development of interventions by the community increased ownership yet never described any engagement efforts.	The intervention was developed to fit communal values by focusing on group-based and sports activities. There is no evidence suggesting the community was involved in defining the problem or identifying the solution. No reflexivity or structural changes are noted.	The intervention only targeted individual behaviour although within a group context. The study did not consider larger systems or environments. Limited systems thinking is evidenced.	Members of an Aboriginal community organisations were consulted although there is not any direct efforts at knowledge translation. The focus of the intervention was on individual behaviour with limited engagement with end users.
Simmons et al. 2008	The engagement aspects included a Māori Steering Group and Māori community health workers. There was not much consultation with the community on the design and implementation of the intervention. CHWs were employed to deliver the intervention though they had no input into the content. The CHWs helped improve the implementation of the intervention.	The community had limited input into defining the problem or solution. The intervention was provided to the CHWs. CHWs helped to adapt research processes to better fit cultural values of the community. CHWs served as personal trainers to participants. There was reflexivity post study although limited evidence during the implementation process.	CHWs had social networks that gave them a unique ability to interact with the participants. The CHWs enhanced the experience for the participant based on their knowledge of the programme and the system. The implementation team did not realise the complex systems elements that shaped CHW work or the social networks of the community that affected implementation of the project (i.e., research model did not fit service expectations of the community).	The research team worked with Māori providers and other end users within the health system to implement the project. However, they were not partners in the project and there is no direct evidence of knowledge translation efforts.
Sinclair et al. 2013	The study was part of an existing CBPR partnership called the PILI ‘Ohana Project. A steering committee assisted in the planning and implementation of this study. Focus groups provided community engagement/perspectives for the design with peer educators used for implementation. Shared decision making and communication reflected throughout the research phases.	The curriculum for this study was adapted for the community using input from the steering committee and community focus group; this ensured the curriculum had culturally relevant knowledge and activities. Peer educators and steering committee members also contributed local and cultural knowledge by reviewing written materials and making suggestions for activities. Reflexivity evidenced through an ongoing partnership.	The partnership involved multiple aspects of the Native Hawaiian health system. Only individual level of analysis for the intervention. Key relationships were built in the community with the project leaders and the local community organisations.	The partnership includes relationship with the Native health system, community health centres, and grassroots organisations. Thus end users were engaged throughout the process. The level of knowledge translation beyond study period is not clear.
Tomayko et al. 2016	Project used CPBR throughout the research process. Community members and tribal leaders were integrated throughout the design and planning of the intervention. Members from health, education, child welfare, and tribal government bodies of the three initial participating communities met with researchers at a collaboration meeting to discuss results from a previous study with the community and possible interventions.	On-going research with the community assisted in building relationships and trust within the communities and study idea came from this previous research so reflects community voice in problem definition and solution. Community organisations were able to adapt the intervention to their communities. All materials and research processes were culturally appropriate (e.g., no control group, but rather having an alternative intervention group and having home mentors be community members).	The intervention focussed on family-level positing the best way to change individual behaviour was to incorporate the family. Other aspects of the community were not directly considered. Home mentors were used to facilitate programme delivery. Other systems thinking aspects were not addressed.	The intervention was developed with key end users including wellness staff and tribal leaders. They offered input to the intervention and the fact that all participants needed to have an intervention. Continued efforts by community to continue obesity prevention efforts included obtaining additional funding.
Qualitative Studies				
English et al. 2008	A CBPR process was used throughout the intervention. Tribal community, academic institution, and intertribal organisations joined together to share information and resources to collectively design a community-based intervention. Many preliminary activities were conducted to build relationships with the community.	The preliminary activities allowed the project team to gain a better understanding of the issues among the community. Education courses were held to advance community empowerment. Focus groups were held for participants to discuss the barriers to receiving health care. Community voice was evidenced through and the partnership used reflexive dialogue. Resources were brought to community members to facilitate screening.	The projected was guided by the socioecological framework. Systems networking allowed structural changes to happen within bigger organisations. Hospital staff agreed to setting specific days aside for the participants which encouraged their participation in the project. Community health workers were members of the community themselves and worked to inform participants and recruit for the project.	Tribal leaders and community organisations were included in the participatory process. Efforts sought to develop policy change. Authors concluded that the intervention was sustained by the community.
Sushames et al. 2017	There is no clear initial consultation with the participants before the intervention was designed and implemented. It was noted there was a participatory process with support by local Indigenous mentors and a local Aboriginal health organisation. Despite this notation, there is no clear indication of shared decision making or partnership between the researchers and the community.	The intervention included interviews although they were only conducted post intervention. They were also conducted by a non-Indigenous researcher, who had previous established relationships with the participants. The analysis was conducted by the research team. No clear evidence of its processes in designing the intervention and inclusion of community voice beyond the use of local Indigenous mentors. The mentors helped provide insights on cultural ideologies.	The study focused on enablers and barriers to participation which included elements at various levels in the system. These helped illustrate the larger community systems and relationships among people and cultural constructs. The study helped to illustrate why the intervention was not well attended despite having positive impacts.	There was limited engagement with end users with only a local organisation consulted primarily for recruiting participants. It was noted that this intervention did not have sustainable outcomes for the communities.
Townsend et al. 2015	The study was part of an existing CBPR partnership called the PILI ‘Ohana Project. An existing steering committee assisted in the planning and implementation of this study. This study involved genetic testing and engagement with the steering committee was paramount for the implementation of the study.	Biospecimens were required for this study which caused some concern for the community. The steering committee met several times to determine an appropriate process. The intervention had semi-structured support groups to address any new or pending concerns from the participants. They also held an informal meeting for community participants for reassurance. Thus, there was good community voice for approval of the project. Reflexivity is evidence by ongoing dialogue about the project.	The project acknowledged the larger history about genetic testing in Indigenous communities and sought ways to address these issues. A follow-up was conducted in a community setting and the committee took on board the feedback from the participants to create a newsletter presenting the general findings. Focus groups were also held with eight participants to discuss their thoughts on the intervention. These enabled feedback loops to be included.	End users from the community organisation where the study took place was engaged in the process. This engagement and trust building allowed the study to happen. Further data collection/genetic testing is thus an option.
Tumiel-Behalter et al. 2011	This study was held in four different communities (one Indigenous). Each community initially received the same core intervention although researchers and community partners quickly learnt that they needed to be adapted to each community. Researchers reached out to community organisations within the areas to identify a community partner to collaboratively adapt and implement the programme.	Community partners and participants were asked to provide feedback to the overall project team to improve the program. Various formats such as focus groups, conversational interviews and surveys were implemented throughout the process on an ongoing basis to continually improve the program. The feedback assisted not only the overall project, it also allowed the community partners to create the changes. Community voice was acknowledged in the adaption of the core programme.	Project staff sought to integrate the intervention into the local community infrastructure. Activities targeted individual and community change. There was acknowledgement of social determinants of health and key social issues (e.g., drugs, violence, and unemployment).	End users in each community were consulted with the goal to initiate the programme with funds, staff and resources, and to gradually transfer ownership and leadership to the community partner as the programme progressed. The input from the end users helped to make the programme sustainable in most of the communities, including the Indigenous community.

For the CCA, there are three key issues to consider: voice/agency, reflexivity and structural change and resources. For voice/agency, there were three patterns identified. The first was studies that reflected community voice/agency in defining the problem and identifying the solution [45, 46, 48–50, 52, 54, 59, 61]. The second was studies that allowed for adaptation of the solution to fit the culture of the community, but without clear choice that this was an important problem to address [51, 53, 56, 58, 62, 63]. The third was studies that did not allow much input into the problem or solution beyond minor changes or simple approval [44, 47, 55, 57, 60, 64]. Reflexivity of the researchers about power relations and relationships among partners was directly expressed by a little more than 40% of the studies [45, 46, 48, 49, 51, 52, 61, 62], although an additional study did include post-study reflection [60]. Finally, while all of the studies offered resources, only a third of the studies sought structural changes through their interventions in the form of changing policies, systems or organisational/community practice [45, 48–50, 58, 59, 61].

High levels of CE were reflected in two thirds of the studies. The most common engagement approach was the use of community-based participatory research (CBPR) which was directly noted by nine of the studies [44–46, 51, 52, 54, 59, 61, 62] with the remaining studies offering another participatory approach [48–50, 53, 63]. Most of these studies involved community partners or steering/advisory groups that guided the work and had shared decision-making and communication responsibilities with the researchers. There was evidence in these studies that the high level of engagement was included throughout the research process from design/adaptation to implementation and evaluation of the intervention. The remaining studies had relatively limited levels of engagement. Some of these would be best described as an initial consultation to get approval for the project with limited input beyond that stage except to help with recruiting participants [47, 55, 57]. Two of these limited engagement studies stated the use of steering/advisory committees to guide the work and yet the evidence is that these groups were primary for consultation and not shared decision-making [56, 60]. Three additional studies stated they used participatory approaches although with limited evidence of who the partners were or how the studies were in fact participatory [44, 58, 64]. Finally, some of these limited engagement studies utilised CHWs to help with engagement with participants even though other aspects of their project were limited engagement [44, 47, 60].

There were three predominant patterns of ST, which in part are based on the level of behaviour targeted and in part on the perspectives and relationships identified. The vast majority of studies targeted individual behaviour (*n* = 16; 76%) with both community and individual-levels targeted by three studies [49, 50, 63] and the community-level only in two studies [48, 55]. The studies that targeted some community-level behaviour represent the first pattern. Each of these studies demonstrated clear understanding of multiple causes and perspectives and included systems-level activities. Most of these studies also had multi-level intervention activities as well. The second pattern was studies that focussed only on individual-level behaviour and demonstrated limited ST [44, 47, 54, 56, 57, 60, 64]. These studies did not integrate multiple perspectives and typically only included minimal feedback loops in adapting the intervention. Two of these studies provided retrospective recognition of ST as providing explanations for the challenges in implementing the intervention [60, 64]. The final pattern were studies that targeted individual-level behaviour although included ST in the design of the intervention. Four of these studies included multi-level activities in the intervention [53, 58, 59, 61], while the others integrated ST through partners and steering committees to help improve implementation effectiveness [45, 46, 51, 52, 62].

IKT includes three predominant approaches in the studies. First, nine studies had limited or no knowledge translation activities or engagement with end users [44, 45, 47, 55–58, 60, 64]. These studies may have consulted end users at the beginning of the study although that was primarily for the purpose of approving the study or gaining access to participants. One of these studies did actively engage with end users in knowledge translations at the end of the study although they did not appear to be integrated throughout the study [58]. Second, four studies included end users through a steering committee that included members of the health system [46, 51, 52, 62]. Thus, the end users were integrated into the design and implementation of the intervention; however, these studies did not directly discuss how knowledge translation activities occurred or whether the intervention was sustainable. Third, eight studies described the integration of community and organisational leaders throughout the design and implementation process and also discussed how the study led to continued activities or funding and/or structural or policy changes [48–50, 53, 54, 59, 61, 63]. These studies represent high levels of IKT.

## Discussion

The purpose of this study was to systematically review the implementation of non-communicable disease health interventions into Indigenous communities and identify the degree to which HPW elements were reflected in these studies. The studies demonstrate a number of positive health outcomes at both individual and community levels and cover a range of non-communicable diseases. Two key patterns emerge about the implementation of these interventions: (a) high levels of CE and CCA—including the prominence of community health workers—and (b) comparatively lower levels of ST and IKT. Implications and limitations are noted.

### Community engagement and culturally-centred approach

About two thirds of studies identified participatory approaches as being prominent in the design. These findings reflect the extant literature that argues for participatory approaches to developing and implementing health interventions with Indigenous communities [23, 65, 66]. Further, a variety of systematic and meta-analytic reviews have found positive associations between CE and health outcomes [21, 26, 67] with the most popular CE approach being CBPR. In addition, an international literature review found that CE has been linked to positive outcomes such as social capital and neighbourhood unity for socially excluded groups [21]. This is supported by an evaluation suggesting that interventions led by community organisations were more successful at engaging secluded groups than government initiatives [67].

Similarly, the CCA is consistent with CE as it emphasises community voice/agency for engaging in change around health [17]. Such an approach centres culture and cultural perspectives and thus is consistent with Indigenous autonomy and self-determination. Many Indigenous organisations have placed a priority on the development of an Indigenous health workforce that has both professional and cultural competence [68], drawing on the fact that culturally adapted health interventions are more effective than traditional “top-down” interventions [69]. Beyond the participatory approach, the CCA advocates for reflexivity of external partners and structural change to facilitate implementation effectiveness. These elements reflect the need for interventions to provide resources and systems change to improve health equity [17]. However, only slightly more than a third of the studies had evidence of research reflexivity or structural change within the studies.

A key way that many studies helped to support CE and/or the CCA was the use of CHW. The majority of studies in this review used CHW although they may have been called lay health advisers, peer educators, or lifestyle coaches. CHW are considered to be successful due to the relationship they have with the community; they are trusted members who are able to communicate effectively with community members because they are aware of cultural values and reflect the diversity of the population served (i.e., they have cultural knowledge) [70]. The prominence of CHW in these studies is consistent with the extant literature finding frequent use of CHW particularly in Indigenous and ethnic minority communities [71–73]. CHW involvement in interventions is associated with a variety of positive health outcomes including non-communicable diseases and benefits to health service utilisation [71, 74]. CHW are also generally part of an overall philosophy that reflects Indigenous knowledge and participatory approaches such as community engagement [75]. However, it is important to note that some studies in this review that have lower CE and CCA still used CHW [47, 60]; hence, the presence of CHW does not mean an intervention automatically has high levels of CE and CCA.

### Systems thinking and integrated knowledge translation

While the reviewed studies collectively had high levels of CE and CCA, there were fewer studies with high levels of ST and even fewer with a high level of IKT. For ST, only a small number of studies had multi-level perspectives and activities and focused on outcomes at a systems level. More studies included information reflecting systems perspectives and multi-level activities although focussed only on individual-level outcomes with a third of studies having limited ST. ST helps to identify a holistic perspective of health issues and also provides boundaries of the intervention within the system for effective implementation (e.g., recognising facilitators and barriers) [76]. Recent literature suggests that combining participatory approaches with ST is the key to improving health equity in communities [29]. Participatory approaches enable multiple stakeholders and perspectives consistent with ST; however, the current review included studies with strong participatory approaches without ST [54] and also strong ST without participatory approaches [55].

Slightly more than a third of the studies in this review demonstrated high levels of end-user engagement and thus IKT, while the other studies had limited or only some engagement with end users. IKT is an important factor for facilitating the translation of evidence-based interventions into policy and practice as it helps to navigate larger health systems and the perspectives of key stakeholders [33, 37, 77]. End users often have the power to shape policy and provide resources to sustain interventions; their integrated engagement provides an opportunity for researchers to understand the larger policy and practice context [33]. Co-design of research between Western researchers and Indigenous end users also facilitates effective knowledge translation between Western scientific and Indigenous knowledge systems [37].

### Implications

This review utilised the HPW framework in a post hoc manner to identify patterns in the implementation of chronic condition health interventions in Indigenous communities. Several implications and some future research results from this review. The relatively high levels of CE and CCA are consistent with autonomy and self-determination in Indigenous communities. Autonomy has not been handed to the Indigenous communities, but rather it has been demanded by many Indigenous cultures as a rejection of policies of assimilation resulting from colonial histories [68]. Self-determination is a key element for implementation and intervention effectiveness in Indigenous communities [15, 65]. Self-determination facilitates acceptability of interventions because it ensures a sense of ownership, cultural relevance and the centring of Indigenous knowledge to the health problem. Self-determination is often achieved through participatory approaches like CBPR because of the shared decision-making in the interventions [78].

Additionally, enhancing ST and IKT may enhance the sustainability of the health interventions. However, there is clearly a need for future research in this area. This review identified a lack of long-term or systems-level outcomes overall. Much of the focus in outcomes was on individual-level behaviour and knowledge changes. Sustainability was often only included in the discussion of the future implications for the research and not within the research project itself; there were certainly exceptions to this with some projects designed around creating an intervention sustainable beyond at least the study period, and these studies reflected high levels of ST and IKT [49, 50].

Very few of the reviewed studies demonstrated high levels of all of the HPW elements. What is not clear at this stage is whether a given intervention needs to be strong in each factor to address health equity. The extant literature demonstrates that each element has positive associations with some aspects of health; although it is not clear whether the collective elements are needed to make a significant improvement in health equity. The diversity of health outcomes, from individual knowledge to system-level change, make it difficult to directly compare the value-added for each individual element. Thus, the current state is that this review provides insights about likely avenues to improve implementation effectiveness for achieving gains towards health equity although does not provide direct evidence.

Finally, there are some interesting insights about the methodological appraisal and the study characteristics. Given that these were studies in the community, most of the studies lacked some of the key elements of traditional research design such as strict blinding in randomisation or even formal adherence to randomisation. Further, in all but two of the randomised trials included in the reviews [53, 60], the intervention was eventually delivered to the comparison group or an alternative intervention was provided. This approach is common in Indigenous communities as to withhold an intervention is not consistent with collective values and inclusiveness [66]. These approaches are ways to decolonise research methods to ensure the Indigenous knowledge and values are strongly reflected in the research [66]. This type of inclusivity and community benefit likely should be included in methodological appraisals of Indigenous community health interventions.

### Limitations

This review is not without limitations. First, given the diversity of health issues, study outcomes and levels of analysis, it is not possible to link study outcomes to specific aspects of the HPW framework. Future research can better explore the concrete relationships between elements of the framework and specific health outcomes. Second, while we have attempted to be rigorous in our search strategy, it is possible that relevant studies have not been included, particularly those not formally published. Also, in the search terms, we opted to use “Native American” and not “American Indian;” hence, relevant studies may have been missed. Finally, we acknowledge that our findings and conclusions are based on the data each publication has provided even if they did not label the information as a particular HPW element. The lack of data regarding ST and IKT does not necessarily mean that they did not consider those elements as page limits may limit reporting of some information. However, we encourage researchers to report on all four HPW elements when describing the implementation of health interventions with Indigenous communities to enable knowledge consolidation about these topics and advance thinking about how best to apply these principles for improved implementation and maximum impact.

## Conclusion

In conclusion, the He Pikinga Waiora Implementation Framework posits that participatory approaches such as CE and CCA, along with ST and IKT, are important to utilise when developing health interventions for Indigenous communities in order to achieve health improvement and health equity. This framework reinforces the idea that Indigenous communities will support health interventions that they help to create as it aligns with their cultural views, making the intervention more beneficial and sustainable for the community. The current review illustrates various patterns of each of the HPW elements with CE and CCA more prominent than ST and IKT. The review also illustrates that few studies incorporate all four elements of the HPW framework although future research is needed to determine the value added for each of the elements.

## Additional files

Additional file 1: PRISMA checklist. (DOC 64 kb)

Additional file 2: Additional references. (DOCX 18 kb)

Additional file 3: Study characteristics. (DOCX 30 kb)

## Data Availability

Articles included in the analysis are cited in the reference list.

## Abbreviations

CCA: Culture-centred approach
CE: Community engagement
CHW: Community health workers
HPW: He Pikinga Waiora (Enhancing Wellbeing) Implementation Framework
IKT: Integrated knowledge translation
PRISMA: Preferred Reporting Items for Systematic Reviews and Meta-Analyses
ST: Systems thinking
USA: United States of America
