# Supplementary material for: Implementation effectiveness of health interventions for indigenous communities: a systematic review
Source: Implement Sci. 2019 Aug 5;14:76. doi: 10.1186/s13012-019-0920-4 (PMC6683565; doi:10.1186/s13012-019-0920-4)
Supplement: Supplementary file 2 — Additional references. (DOCX 18 kb) [file 13012_2019_920_MOESM2_ESM.docx]

**Additional File 2. References Consulted Related to Primary Study and Quality of Final Details on Intervention Development and Implementation Related to He Pikinga Waiora Elements**

| **Primary Study** | **Additional References Consulted** | **Details (Good, Fair, Poor** |
| --- | --- | --- |
| **Observational Studies** | | |
| Benyshek et al. 2013 | None | Fair |
| Christopher et al. 2008 | Smith A, Christopher S, Knows His Gun McCormick A. Development and implementation of a culturally sensitive cervical health survey: A community-based participatory approach. Women Health. 2004; 40(2): 67-86. | Good |
| Coppell et al. 2009; | Tipene-Leach DC, Coppell KJ, Abel S, Pāhau HL, Ehau T, Mann JI. Ngāti and healthy: translating diabetes prevention evidence into community action. Ethn Health. 2013; 18:402-14. | Good |
| Kaholokula et al. 2014 | Mau MK, Kaholokula JK, West M, et al. Translating diabetes prevention into Native Hawaiian and Pacific Islander communities: the PILI 'Ohana Pilot project. Prog Community Health Partnersh. 2010; 4: 7-16; Nacapoy AH, Kaholokula JK, West MR, et al. Partnerships to address obesity disparities in Hawai'i: the PILI 'Ohana Project. Hawaii Med J. 2008; 67(9): 237-41. | Good |
| Kakekagumick et al., 2013 | Saksvig BI, Gittelsohn J, Harris SB, Hanley AJ, Valente TW, Zinman B.A pilot school-based healthy eating and physical activity intervention improves diet, food knowledge, and self-efficacy for native Canadian children. J Nutr. 2005; 135:2392–8. | Good |
| Reilly et al. 2011 | Reilly R, Doyle J, Rowley K: Koori community-directed health promotion in the Goulburn Valley. Australian Community Psychologist. 2007; 19:39-46. | Good |
| Shah et al. 2015 | Supplemental File: Study Protocol | Good |
| **Randomised Control Trial** | | |
| Brimblecombe et al. 2017 | Brimblecombe J, Ferguson M, Liberato SC, et al. Stores Healthy Options Project in Remote Indigenous Communities (SHOP@RIC): a protocol of a randomised trial promoting healthy food and beverage purchases through price discounts and in-store nutrition education. *BMC Public Health* 2013; 13: 744. | Good |
| Canuto et al 2012 | Canuto K, McDermott RA, Cargo M, Esterman, AJ. Study protocol: a pragmatic randomised controlled trial of a 12-week physical activity and nutritional education program for overweight Aboriginal and Torres Strait Islander women. BMC Publ Health 2011, 11:655. | Good |
| Ho et al. 2008 | Rosecrans A, Gittelsohn J, Ho LS, Harris SB, Ford E, Naqshbandi M, et al. Process evaluation of a multi-institutional community-based program for diabetes prevention among First Nations. Health Educat Res. 2008: 23: 272-86. | Good |
| Kaholokula et al. 2012 | Mau MK, Kaholokula JK, West M, et al. Translating diabetes prevention into Native Hawaiian and Pacific Islander communities: the PILI 'Ohana Pilot project. Prog Community Health Partnersh. 2010; 4: 7-16; Nacapoy AH, Kaholokula JK, West MR, et al. Partnerships to address obesity disparities in Hawai'i: the PILI 'Ohana Project. Hawaii Med J. 2008; 67(9): 237-41. | Good |
| Karanja et al. 2010 | Supplementary appendix | Fair |
| Kolahdooz et al. 2014 | Sharma S. Assessing diet and lifestyle in the Canadian Arctic Inuit and Inuvialuit to inform a nutrition and physical activity intervention programme. J Hum Nutr Diet. 2010; 23:5–17; Sharma S, Gittelsohn J, Rosol R, Beck L. Addressing the public health burden caused by the nutrition transition through the Healthy Foods North nutrition and lifestyle intervention programme. J Hum Nutr Diet. 2010; 23:120–28 | Good |
| Mendham et al. 2015 | None | Fair |
| Simmons et al. 2008 | Blundell R, Gibbons V, Lillis S. Cultural issues in research, a reflection. N Z Med J. 2010; 123(1309):97-105. | Good |
| Sinclair et al. 2013 | Nacapoy AH, Kaholokula JK, West MR, et al. Partnerships to address obesity disparities in Hawai'i: the PILI 'Ohana Project. Hawaii Med J. 2008; 67(9): 237-41. | Good |
| Tomayko et al. 2016 | Adams AK, LaRowe TL, Cronin KA et al. The Healthy Children, Strong Families intervention: design and community participation. J Prim Prev. 2012; 33: 175–85. | Good |
| **Qualitative Studies** | | |
| English et al. 2008 | None | Good |
| Sushames et al. 2017 | None | Fair |
| Townsend et al. 2015 | Nacapoy AH, Kaholokula JK, West MR, et al. Partnerships to address obesity disparities in Hawai'i: the PILI 'Ohana Project. Hawaii Med J. 2008; 67(9): 237-241. | Good |
| Tumiel-Behalter et al. 2011 | None | Good |
