# Supplementary material for: Implementation effectiveness of health interventions for indigenous communities: a systematic review
Source: Implement Sci. 2019 Aug 5;14:76. doi: 10.1186/s13012-019-0920-4 (PMC6683565; doi:10.1186/s13012-019-0920-4)
Supplement: Supplementary file 3 — Study characteristics. (DOCX 30 kb) [file 13012_2019_920_MOESM3_ESM.docx]

**Additional File 3. Study Characteristics**

| **Study** | **Participants** | **Intervention** | **Methods** | **Study Findings** | **≥1 Primary Effect** | **50% of Effects** |
| --- | --- | --- | --- | --- | --- | --- |
| **Observational Studies** | | | | | | |
| Benyshek et al. 2013 | 22 American Indian/Alaska Natives living in Las Vegas, Nevada, USA | 16-week type-2 diabetes prevention curriculum with weight-loss curriculum, meal planning, fat gram and calorie counting, portion size, and food content; delivered by lay lifestyle coaches | Pilot, single group pre- and post-study design; Baseline and post-participation survey along with clinical measures using standardized protocols;12 completers (55%), 3 partial completers (no clinical measures), 7 non-completers (only baseline)—used intention-to-treat analysis; inclusion/exclusion criteria of Body-Mass Index (BMI) ≥25 and HbA1c (between 5.4% and 6.4%; 36-46 mmol/mol) with no major illness or using medication that would interfere with glucose tolerance; no random selection | Weight loss (-5.79%, p=.01); BMI (-5.9%, p=.01; Waist circumference (-4.34% reduction, p=.01); Triglycerides (-15.89% reduction, p=.71); HDL cholesterol (+12.92%, p =.007), Fasting blood glucose (-.39%, p=.50); Systolic BP (-6.04%, p=.34), Diastolic BP (-1.95%, HbA1C (no change); Qualitative results identified 8 themes related to program experience | Y | N |
| Christopher et al. 2008 | 101 women in the Apsáalooke community, Montana, USA | Cervical cancer education and general health education delivered by lay health advisors (Messengers for Health) | Single group pre-test/post-test design; three-year follow-up; 83 completers of both tests (82%); Used scales adapted from other surveys to measure pap test knowledge, cervical cancer knowledge, comfort discussing cancer issues, and awareness—internal consistency was assessed no validity information provided; Random selection of participants | Pap test knowledge (-.11, p=ns); Cervical cancer knowledge (+.29, p<.05); Comfort (+.42, p<.05); Awareness of cervical cancer-2 questions (.16, p< .01; .24, p<.001, .70); Qualitative results found support for using of community-based participatory research (CBPR) approach; the program was widely accepted | Y | Y |
| Coppell et al. 2009 | 286 at baseline and 235 at follow-up in a predominantly Māori community from the East Coast (Gisborne), New Zealand | Community-led diabetes prevention program (Ngati and Healthy) aimed at the entire community; Involved local health promotion programs, community education program for high-risk individuals, and a structural strategy. Used community health workers | Interrupted time-series prevalence surveys; Two-year time difference between two separate randomly selected samples; Exclusion criteria were terminal illness, death or migration form study area; Used clinical and validated self-reported measures | Selected findings: Insulin resistance (-10.1%, p=.0003); Among 25-49 women: 5+ times/week exercise (+15.16%, p=.04); Wholemeal bread (+20.3%, p=.0002); Among 25-49 men (-.15mmol/l HDL cholesterol, p=.038) | Y | N |
| Kaholokula et al. 2014 | 239 Native Hawaiian and Pacific Islander adults in Hawaii, USA | Three-month (16 lessons) adapted diabetes prevention program; Delivered by community health advocates; Adapted CBPR | Single group pre-test/post-test design with four different community groups receiving same intervention; Inclusion criteria was 18 years of age or older, ethnicity, BMI ≥ 25 (≥ 23 for Filipinos), have a family member or friend to participate; Measures included clinical (anthropometric), behavioural, and demographic as confounders; Measures were reliable and valid | Weight (-1.7 kg, p <.001); BMI (-0.6, p<.001), Systolic BP (-3.3, p<.01), Diastolic BP (-3.4, p<.001), 6-minute walk test (+106.6 ft walked, p<.001), Physical activity frequency (+0.5, p<.01), Fat in diet (-0.3, p<.001); Comparisons of the four groups showed differences in outcomes with Native Hawaiians fairing better than immigrant Pacific Islanders | Y | Y |
| Kakekagumick et al., 2013 | Members of the Sandy Lake First Nations community, Canada including 47 students | Sandy Lake Health and Diabetes project to address type-2 diabetes and including multiple elements over a 20 year period: community survey, food store program, home visit program, diabetes road show, and school-based curriculum; Delivered by various community workers | Study reports results from school-based curriculum that included a pre-test/post-test design with four data collection points over 8-month period; Measures included self-report student questionnaire, 24 hour diet recall, anthropometric data, and physical activity test; 80% retention rates over four time periods; Details about participants are missing | Self-efficacy increased from initial to final (=0.4, p<.001) along with health and dietary knowledge (+0.21, p<.001); Time watching TV decreased (-62 minutes/week, p<.05); BMI increased (+2.7, p<.0001) | Y | N |
| Reilly et al. 2011 | 1800 Aboriginal people in three community organisations of northern Victoria, Australia | Health promotion program implemented by local health workers; Included health summer school for practitioners, nutrition program for under 17-footballers; initiatives aimed at improving dietary quality at a football/netball club; focus groups for adapting nutritional guidelines; weekly self-directed meeting for women; and workplace exercise program | Ecological analysis of the health program using a scoring framework; Included store turnover of the football/netball club; Questions adapted from prior studies to evaluate each of the activities (validity and reliability of measures unclear); Outcomes were at organisational level; One year follow-up although results presented only at a descriptive level | Food store turnover: Increase in fresh meat, eggs, fruit, vegetables and bread/flour with decreases in cakes, confectionary and pies; Ecological analysis: 10 different activities targeted organisations and individuals although no specific outcomes provided | Y | Unknown (total number of effects not clear) |
| Shah et al. 2015 | 60 Zuni (American Indians) with type-2 diabetes in New Mexico, USA | Home based intervention delivered by community health representatives to improve self-managed care; Included one hour didactic and then monthly educational classes of 7-10 individuals | Single group pre-test/post-test design; Measures included physical exam, point of care testing, educational intervention survey and the patient activation measure; Six- month follow-up; Inclusion criteria included HbA1C > 6.5%; No drop-outs | Patient activation increased by one level in 58% of patients, 40% did not change and 2% declined one level; HbA1C (-.73, p=.001), fasting blood glucose (-23.8 mg/dl, p=.0003), BMI (-1.4, p=.001 ) total cholesterol (-11.5 mg/dl, p=.003), triglycerides (-38 mg/dl, p=.001) | Y | Y |
| **Randomised Control Trial** | | | | | | |
| Brimblecombe et al. 2017 | 20 communities in remote Indigenous communities, Northern Territory, Australia | 20% price discounts on food and drink purchases with and without consumer education to determine impacts on fruit and vegetables purchased; Consumer education included monthly messages for six months; Collaborated with retail store associations and community leaders | Stepped-wedge randomised trial with randomisation at the community level and stratified by store association to receive consumer education (n=10) or not (n=10); Inclusion criteria was community with at least 100 people, very remote and socioeconomic disadvantaged and community store managed by one of two associations with no other store within 20km; Weekly store sales data in 20 stores were collected 49 weeks baseline; 24 week intervention phase and 24 week post intervention on fruit and vegetables, drinks and other foods; Sensitivity analysis was conducted by removing one store at a time in the analysis | Price discount was associated with a 12.7% (p<.001) increase in purchases in grams of fruit and vegetables during the intervention and a 19.8% (p< .001) increase post-discount; Consumer education had no significant impact on combined fruit and vegetable purchases or on most purchases--it did have an impact on vegetable purchases only during the discount (+13.6%, p =.014) and not after (+9.1%, p =.055). | Y | N |
| Canuto et al. 2013 | 100 Aboriginal and Torres Strait women aged 18-64 in Adelaide, Australia | 12-week exercise and nutrition program including two 60-minute group exercise classes/week with a fitness instructor and four nutrition workshops with dietician overall; Constructed with consultation from two community organisations and advisory group | Participants were randomly assigned to an active or waitlisted (comparison group); Inclusion criteria: waist circumference > 80; Exclusion criteria were pregnancy and physically unable to participate; Measures included anthropometrics and clinical measures such as HbA1C, lipid profile, blood pressure prior to the program, immediately after the program and then with additional 3-month follow-up; 59% retention rate; Analysis controlled for baseline demographics and lost to follow-up included | Active group, compared to waitlisted group, had significant reductions in weight (1.65 T2 and 2.5 T3) and BMI (.66 T2 and 1.03 T3); Waist circumference and clinical measures were not significantly changed | Y | N |
| Ho et al. 2008 | 133 First Nations people in Ontario, Canada | 6-8 week school, store and community intervention for prevention of diabetes risk factors (Zhiiwapenewin Akino’maagewin: Teaching to Prevent Diabetes); Improve food options at stores; Community events and health promotion; Delivered by a trained program assistant who was a community member | Quasi-experimental pre-test/post-test design; Intervention group at the community level; 2 matched intervention and 2 matched control groups; Exclusion criteria included pregnancy, recent birth or not living in community for 30 days; 9 month follow-up period with 71% retention; Measures include self-reports of food knowledge and healthy food behaviours with mixed reliability quality; Anthropometric measures assessed with standard equipment; Confounding measures included gender, age, and socio-economic status | Food knowledge (β=.10, p=.02) and health food acquisition (β=.95, p=.003) higher for intervention group; No difference in BMI (β=.82, p=.11) and body fat % (β=.15, p=.89); No difference in physical activity | Y | N |
| Kaholokula et al. 2012 | 144 Pacific Islanders who had completed a three-month weight loss program (50% Native Hawaiian), USA | PILI Lifestyle Program (PLP), a six-month weight loss maintenance intervention; Monthly sessions; Delivered by trained peer educators; Developed through CBPR process | Pilot randomized control trial with the program compared to a standard program; Eligibility was completion of the initial weight-loss program, BMI > 25, willingness to participate and include at least one family member; Compared people who did not advance to maintenance program and found no difference than those who participated; six-month follow-up with 70% retention; Loss to follow-up included in intention to treat | PLP participants were 2.5 times more likely to maintain weight loss compared to standard program (p=.09); Those who completed at least half the sessions were 5.1 times more likely to maintain weight loss (p=.02) | Y | Y |
| Karanja et al. 2010 | 205 American Indian families from three tribes in the Portland Area Indian Health service, USA | Community-wide intervention plus a family component to promote breastfeeding and reduce the consumption of sugar-sweetened beverages; final goal of reducing BMI-Z scores in children 18-24 months of age; Family component included 7-21 home visits completed by community health workers; Community component include awareness and health education | Three tribes randomly assigned to two active interventions (community intervention; community plus family) in a pre-test/post-test design; No active control group—used pre-test sample of children born two year earlier in the same tribes; Inclusion criteria were family with expectant mother from one of three tribes; 24-month follow-up with 86% completion rate; Measures included chart review of breastfeeding, self-report for confidence and calibrated scales and stadiometers for BMI | Breasting feeding initiation and 6-month duration increased 14 and 15% over national rates; BMI-Z scores decreased in the community plus family intervention compared to community intervention only (-0.75, p = .02) | Y | Y |
| Kolahdooz et al. 2014 | 332 Inuit and Inuvialuit adults living in Artic Canada | 12-month Healthy Foods North intervention program to improve diet; Program including health promotion and educational activities in media, grocery stores, health clinics and community events | Quasi-experimental, pre-test/post-test design of randomly selected participants; Four communities received the intervention and then two control communities received it later; Exclusion criteria were pregnant and lactating women and < 19 year of age; Validated food frequency and adult impact questionnaires along with anthropometric measures were used; One-year follow-up period Retention rates of at least 83% | For intervention compared to control, decrease in high fat meats (-27.9g, p<.05) and high fat dairy (-19.8g, p<.05) and increase in healthier preparation methods (0.5, p<.001) | Y | N |
| Mendham et al. 2015 | 33 inactive Indigenous Australian men, New South Wales | 12-week sports based exercise intervention for markers associated with type-2 diabetes; Weekly training for 2-3 days in a group environment; Supervised by a fitness instructor | Pilot randomized control trial with exercise (n=16) and control (n=10) groups in a pre-test/post-test design; Exclusion criteria was a diabetes diagnosis; Measures included glucose regulation, anthropometrics, and inflammatory markers and peak aerobic capacity; 64% retention rate with three-month follow-up; Lost to follow-up not included in final analysis | Exercise condition decreased insulin resistance, insulin area under the curve, BMI, waist circumference, waist to hip ratio and increased estimated insulin sensitivity and peak oxygen consumption compared to control group (p<.05) | Y | N |
| Simmons et al. 2008 | 160 Māori without diabetes in New Zealand | Personal trainer who was a Māori community health worker to help prevent progression of impaired glucose tolerance to type 2 diabetes; Workers followed structured interview approach with patients received baseline results for tailored advice on weight loss | Pilot randomized control with pre-test and post-test design (part of larger cluster-control trial); Participants compared to 52 weighed immediately before intervention and 1143 people from the same geographical area; Measures included clinical and anthropometric although only weight presented in this study; Approximately 12-month follow-up (not directly reported) and retention rates of 66%; those lost to follow-up not discussed or included in analysis | Most participants (n=106) had significant weight loss from first to last visit; those with IGT (n=27) experienced significant weight loss at final visit (5.2 kg, p<.01); No significant difference between treatment and control group at first visit (only direct comparison made) | Y | N |
| Sinclair et al. 2013 | 82 Native Hawaiian and Pacific Islanders with diabetes, USA | Culturally adapted diabetes self-management program (Partners in Care, PIC); Used CBPR methods to adapt the program; Community peer educators helped adapt the program, recruited participants and delivered the program | Pilot test randomised control with pre-test and post-test evaluation (n=48 treatment and n=34 control); 3-month follow-up with 71% retention for treatment and 91% for control; Inclusion criteria of ethnicity, 18 years or older, had type-2 diabetes, and HbA1c≥7; Measures of HbA1C and self-report of understanding self-management, self-care activities, and distress; Used intent to treat and completed case analysis | Significant difference from intention to treat in HbA1C (-1.1, p<.0001), understanding (+13.1, p<.0001), and performing self-management (+4.9, p<.0001) | Y | Y |
| Tomayko et al. 2016 | 150 adult/child (2-5 years old) dyads from families in four American Indian communities in Wisconsin, USA | Family-based toolkit to address obesity; 12 lessons delivered via community-based home mentor or monthly mailings; Home mentor a member of the community; Developed through a CBPR approach | Randomly assigned families to one of two treatment arms (home-based mentor or mailings) with pre-test/post-test design (eight families shifted to mailing arms after randomisation in alignment with CBPR principles); Inclusion criteria were families with child aged 2-5 years old without major behavioural or physical problems; Clinic sample was used as a comparison group; Primary measures included child and adult BMI and secondary measures included fruit/vegetable consumption, sugar consumption, television viewing, physical activity, self-efficacy and perceived health status; Two-year follow-up with 65% overall retention rate; Multiple imputation used for data from lost to follow-up | No significant effect of the treatment arms; Both arms showed improvements in the following: child BMI percentile (p<.05), child fruit/vegetable consumption (p<.05), child television viewing (p=.05), adult television viewing (p=.002), adult self-efficacy (p=.006) and quality of life (p=.02); No change found for adult BMI | Y | N |
| **Qualitative Studies** | | | | | | |
| English et al. 2008 | 39 women 50 years and older of the Ramah Band of Navajo Indians, New Mexico, USA | A program of individual, community and environmental elements to increase mammography rates (Ramah Navajo Mammography Days); Community health representatives participated in multiple aspects | Used CBPR principles to co-design the program; Conducted focus groups to identify key factors for early breast cancer detection; Included a short- self-report survey questionnaire about mammograms and attitudes post the events; Thick description of the phases of research provided | Focus groups identified knowledge, attitudes and beliefs that were used in the design of the intervention; 36% of the women received their first mammogram ever; 82% hadn’t had a mammogram in at least five years prior to participating; 100% indicated a desire to participate again and would recommend to family and friends. | Y | Unknown (total number of effects not clear) |
| Sushames et al. 2017 | 34 Aboriginal or Torres Straight Islanders in a rural or regional community in North Queensland Australia | Eight-week physical activity program that aimed to improve health outcomes; Delivered by an sport scientist; Intervention was developed using CBPR | Inclusion criteria for the program were ethnicity, aged 18-45 and having a chronic disease or risk of chronic disease; Study used semi-structured interviews to explore the enablers and barriers to participation as the program has low attendance rates; Interview framework loosely guided by Health Belief Model; Interviews conducted by non-Indigenous researcher | Positive attitudes and high levels of motivations; Enablers were participation of family members, no financial cost and a good relationship with the principal investigator; Barriers included work commitments, travel away from community and lack of infrastructure | Unknown | Unknown(no direct outcomes stated) |
| Townsend et al. 2016 | 65 Native Hawaiians with type-2 diabetes, USA | Three-month diabetes self-management program developed through CBPR approach; Delivered by community health workers | Research process was developed through CBPR processes to explore issues of trust; Subset of 16 provided peripheral blood mononuclear cells to investigate molecular mechanisms; A focus group with this subset explored trust issues; Direct quotes from participants not provided | Biospecimen collection in Indigenous communities requires trust of the researchers; CBPR is a key approach for building trust and providing communities voice and protections; Preliminary results indicate changes in DNA that show why the intervention improved HbA1C | Y | N/A (total number of effects not clear) |
| Tumiel-Behalter et al. 2011 | Nearly 3,500 participants in western New York, USA including 563 from the Seneca Nation of Indians | Community program to improve health of four underserved communities (Good for the Neighborhood); Core program includes health screenings, risk assessments, health education and exposure to health services; Delivered by staff of an independent community agency | The purpose was to describe the participatory approach used to develop the intervention; Multi-methods included key informant interviews, focus groups and surveys; Direct quotes from participants not provided | Programs has been sustained for three years and has reached 3,500 participants with 1/3 engaging regularly; Program adapted to focus on educational program, fitness classes, and nutrition classes on the Seneca community | N/A | N/A (total number of effects not clear) |
